# Apoptosis Induction byHistone Deacetylase Inhibitors in Cancer Cells: Role of Ku70

**DOI:** 10.3390/ijms20071601

**Published:** 2019-03-30

**Authors:** Ping Gong, Yuetong Wang, Yongkui Jing

**Affiliations:** Department of Pharmacology, Shenyang Pharmaceutical University, Shenyang 110016, China; gongping1125@126.com (P.G.); 13940326908@163.com (Y.W.)

**Keywords:** HDAC inhibitors, Ku70, apoptosis, Bax, c-FLIP, cancer

## Abstract

Histone deacetylases (HDACs) are a group of enzymes that regulate gene transcription by controlling deacetylation of histones and non-histone proteins. Overexpression of HDACs is found in some types of tumors and predicts poor prognosis. Five HDAC inhibitors are approved for the treatment of cutaneous T-cell lymphoma, peripheral T-cell lymphoma, and multiple myeloma. Treatment with HDAC inhibitors regulates gene expression with increased acetylated histones with unconfirmed connection with therapy. Apoptosis is a key mechanism by which HDAC inhibitors selectively kill cancer cells, probably due to acetylation of non-histone proteins. Ku70 is a protein that repairs DNA breaks and stabilizes anti-apoptotic protein c-FLIP and proapoptotic protein Bax, which is regulated by acetylation. HDAC inhibitors induce Ku70 acetylation with repressed c-FLIP and activated Bax in cancer cells. Current studies indicate that Ku70 is a potential target of HDAC inhibitors and plays an important role during the induction of apoptosis.

## 1. Introduction

Aberrant acetylation of histone and/or expression of histone deacetylases (HDACs) exist in various solid and hematologic malignancies and contribute to tumorigenesis [1]. Overexpression of individual HDACs accelerates the proliferation of tumor cells and predicts poor prognosis in many types of cancer patients [2,3,4,5,6,7,8]. Genetic knockdown of an individual HDAC, most notably HDAC1, 2, 3, and 6, in different types of tumor cells, such as Hodgkin’s lymphoma, gastric cancer, prostate cancer, colorectal cancer, pancreatic cancer, and ovarian cancer, induced DNA damage, cell cycle arrest, and/or apoptosis [9]. HDACs are being considered as therapeutic targets for cancer treatment. An array of natural and synthetic compounds inhibiting HDAC activity, referred to as HDAC inhibitor (HDACi), have been developed [10,11,12]. Although increased histone acetylation by HDACi treatment has been observed, this phenomenon could not be linked to therapeutic outcome. A series of non-histone proteins including p53, heat shock protein 90 (Hsp90), Akt, tubulin, nuclear factor-k-gene binding (NF-κB), signal transducers and activators of transcription 3 (STAT3), and many others, are also substrates of HDACs [13,14,15]. Cell cycle arrest, cell death, differentiation, autophagy, and senescence account for the antitumor effects of HDACis [10,12]. HDACis induce apoptosis in many types of cancer cells and show relative selectivity to malignant cells. Apoptosis appears to be a key mechanism by which HDACis exert therapeutic effects [16,17,18,19]. Recent studies have shown that HDACi-induced apoptosis involves acetylation of ku70 [20]. The development of HDACis and the potential roles of Ku70 in the HDACi-induced apoptosis are summarized.

## 2. Classification of HDACs and Cellular Location

Eighteen HDAC subtypes have been found in mammalian cells and are divided into four classes. Class I, II, and IV comprise the “classical” Zn^+^-dependent HDACs while class III is an NAD^+^-dependent sirtuin [21,22]. Class I HDACs contain HDAC1, 2, 3, and 8. HDAC1, 2, and 8 are localized in the nucleus while HDAC3 is localized in both the nucleus and cytoplasm. Class II HDACs includes class IIa (HDAC4, 5, 7, 9) and class IIb (HDAC6, 10). Class IIa HDACs shuttle between the nucleus and the cytoplasm when stimulated by different signals while class IIb HDACs is mainly localized in the cytoplasm. Class IV HDACs contain only HDAC11, which has similar features as class I and class II HDACs and is localized in both the nucleus and cytoplasm. Class I HDACs distribute throughout the body, while class II and IV HDACs exhibit tissue-specific expression and distribution in smooth muscle, heart, brain, liver, and colon [23]. Class III HDACs contain seven sirtuin proteins (SIRT1–7) that are expressed in the nucleus, cytoplasm, and mitochondria. Class III HDACs do not share homology with other classes HDACs and are not inhibited by generic HDAC inhibitors [24,25].

## 3. Biological Functions of HDACs

Deacetylation of histones has been considered as the main function of HDACs. In eukaryotic cells, chromatin is a dense and higher-order structure consisting of DNA and core histone proteins. The nucleosome formed by histone octamers encircling the DNA superhelix is the elementary unit of chromatin [26]. HDACs do not directly bind to DNA but function in transcriptional corepressor complexes with Sin3, nucleosome remodeling deacetylase (NuRD), REST corepressor (CoRest), nuclear receptor co-repressor (N-CoR), and silencing-mediator of retinoic and thyroid receptors (SMRT) [27]. HDACs catalyze deacetylation of the N-terminal lysine residues in core histones and confer a positive charge that enhances the affinity of histones for the negatively charged DNA. Nucleosome histone protein H2A, H2B, H3, and H4 are substrates of HDACs. Hypoacetylation of histones causes chromatin aggregation, which prevents the transcriptional machinery from being close to the DNA and represses gene transcription [28].

**Figure 1:**
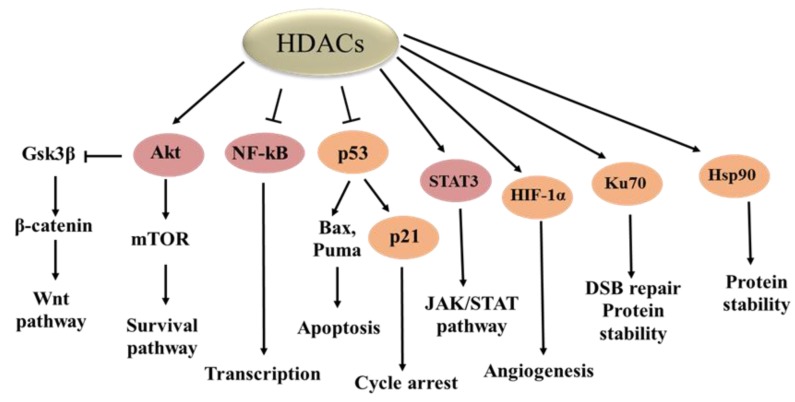
Non-histone protein substrates of histone deacetylases (HDACs) regulate tumor cell proliferation, cycling, apoptosis, and DNA damage repair. GSK3β, glycogen synthase kinase3β; DSB, double-strand break.

HDACs also regulate deacetylation of multiple non-histone protein substrates and impact their functions by altering their activity, cellular location, and protein–protein interactions. More than 50 non-histone proteins have been reported to be the substrates of HDACs. Representatives of non-histone proteins include several important factors, such as p53, NF-κB, STAT3, Hsp90, Akt, and Ku70 (Table 1) [29], which regulate cellular development, proliferation, differentiation, and death and are involved in tumorigenesis (Figure 1).

p53 is a tumor suppressor, and its activity is regulated by acetylation [30]. p53 acetylation blocks ubiquitination and enhances protein stability. Acetylated p53 is an active form and regulates the expression of multiple genes related to cell cycle and apoptosis [30,31,32]. HDAC complexes containing HDAC1 or SIRT1 deacetylate p53 and reverse p53-mediated gene transcription [33,34,35]. HDAC2, 3, 6, and SIRT2 also deacetylate p53 and repress its function [35,36]. p21^cip1/waf1^ is a transcription target of p53, which can be induced in p53-dependent and independent ways in HDACi-treated cells. p53 has been found to play an important role in HDACi-induced autophagy and apoptosis [37].

NF-κB is a heterodimer of p65 (RelA) and p50 proteins, an inactive complex present in the cytoplasm due to binding to inhibitory proteins known as IκBs [38]. There are multiple acetylation sites on p65 and that acetylation of p65 changes the cellular location of NF-κB [39]. HDAC3 deacetylates p65 and subsequently results in nuclear export to inactivate NF-κB through binding to IκBα [40]. However, different reports show that HDAC3 also positively modulates NF-κB activity by keeping NF-κB in a deacetylated state [41,42,43]. The difference may rely on distinct acetylation sites, which need to be further identified.

Hsp90 serves as a chaperone protein for proper folding and maintenance of a number of proteins including BCR-ABL, mutant Fms-like tyrosine kinase 3 (FLT3), c-Raf, and Akt [44,45,46,47,48]. Hsp90 is usually maintained in a deacetylated form by HDAC6. HDAC6 knockdown or inhibition induces Hsp90 acetylation and results in degradation of the client proteins [49]. HDACi LBH-589 induces Hsp90 acetylation by inhibiting HDAC6 and leads BCR-ABL to dissociation and degradation in leukemic K562 cells [50]. It has been found that knockdown of HDAC3 results in acetylation of Hsp90 in the nucleus and dissociation of DNA damage response (DDR) and homologous recombination (HR) proteins from Hsp90 [51].

The signal transducers and activators of transcription (STATs) belong to a family of cytoplasmic transcription factors and are regulated by phosphorylation and acetylation. Phosphorylated STAT3 translocates to the nucleus to induce gene transcription and to promote cell–cycle progression [52,53]. Acetylated STAT3 locates in the cytoplasm and competitively inhibits nuclear translocation of the phosphorylated form. HDAC3 can bind to and deacetylate STAT3. HDAC3 inhibition increases cytoplasmic acetylation of STAT3 and decreases p-STAT3 protein in the nucleus [54].

Akt is a key factor of the phosphoinositide 3-kinase (PI3K) pathway. Akt could be acetylated by the histone acetyltransferases (HATs) p300 and P300/CBP-associated factor (PCAF) in tumor cell lines [55]; deacetylation of Akt by HDACs is required to maintain its activity. Inhibition of SIRT1 or SIRT2 causes Akt acetylation and, in turn, blocks Akt phosphorylation (active state) [56]. Inhibition of HDAC3 or HDAC6 facilitates Akt acetylation and reduces the level of phosphorylated Akt [57,58].

## 4. HDACi for Cancer Therapy

A group of small-molecular HDACis has been developed as cancer therapeutics [82]. Four compounds have been approved by the USA FDA and one compound by the China FDA for the treatment of lymphoma and myeloma (Table 2). Vorinostat (SAHA) was the first HDACi approved by the USA FDA for the treatment of cutaneous T-cell lymphoma (CTCL) in 2006 [83]. Romidepsin (FK-228), a natural extraction product derived from *Chromobacteriumviolaceum,* was subsequently approved in 2009 for the treatment of CTCL and peripheral T-cell lymphoma (PTCL) [84]. Belinostat (PXD-101) was later approved in 2014 for the treatment of relapsed and refractory PTCL [85], and panobinostat (LBH-589) was approved in 2015 for the treatment of relapsed and refractory multiple myeloma [86]. Chidamide (CS055/HBI-8000) was approved by the China FDA in 2015 for the treatment of relapsed or refractory PTCL [87]. SAHA, Belinostat, and LBH-589 are pan-HDACis. Romidepsin is a selective inhibitor of class I HDACs. Chidamide selectively inhibits class I HDACs and HDAC10. None of the five inhibitors inhibit the family of SIRTs and none have been extended to treat other types of cancer. These inhibitors have obvious side effects and toxicities that may restrict their use [88,89,90]. Belinostat and Panobinostat show positive reactions in the Ames mutagenic test, while FK-228 and chidamide have no significant mutagenic toxicity [91,92]. It seems that selective inhibition of class I HDACs is less toxic than inhibition of all HDACs. Specific HDAC inhibitors are being developed [10,93,94,95,96]. Class I/II HDACi Ricolinostat (ACY-1215) and Domatinostat (4SC-202); class I HDACi Mocetinostat (MGCD0103), Entinostat (MS-275), and Banatinostat (CHR3996); and class IIb HDACi Abexinostat (PCI-24781) are being tested in the clinic. To improve the therapeutic effects of HDACis, combinations with radiotherapy, topoisomerase inhibitors, proteasome inhibitors, and BET inhibitors are being tested [11,96].

## 5. Apoptosis Induction by HDACis

Several mechanisms including cell cycle arrest, differentiation, autophagy, and apoptosis have been reported to mediate the therapeutic effects of HDACis [8,10]. Apoptosis is found to be the most effective manner of killing tumor cells by HDACi [8,97,98,99].

Apoptosis is mediated by extrinsic and intrinsic pathways. The extrinsic pathway is initiated by the binding of death receptors (DR) to specific ligands such as Fas ligand (FasL) and TNF-related apoptosis-inducing ligand (TRAIL). FasL and TRAIL binding leads to trimerization, followed by the recruitment of multiple factors to form a receptor polymer. The adaptor protein Fas-associated death domain protein (FADD) and caspase-8/-10 are recruited to the intracellular tails of the polymer, forming the death-inducing signaling complex (DISC). Caspase-8 and -10 within the DISC facilitate self-activation and activate caspase-3, 6, and/or 7 to trigger apoptosis [100]. FADD-like interleukin 1β-converting enzyme (FLICE) inhibitory protein c-FLIP, which competitively binds to FADD and inhibits caspase-8 activity, is a key negative regulator protein of the extrinsic apoptosis pathway [101]. The intrinsic pathway is controlled mainly by the Bcl-2 family including pro- and anti-apoptotic Bcl-2 family proteins. Anti-apoptotic Bcl-2 proteins include A1, Bcl-2, Bcl-xL, Bcl-w, and Mcl-1. Pro-apoptotic BH3-only protein Bad, Bid, Bim, Bmf, Puma, and Noxa can inhibit anti-apoptotic proteins or activate the effector proteins Bak and Bax [102]. Activated Bak and Bax oligomerize and form membrane pores in the outer membrane of mitochondria, releasing cytochrome c and Smac/Diablo into the cytoplasm, leading to the activation of caspase-9 and caspase-3 and inducing apoptosis [103].

HDACis modulate the balance between pro- and anti-apoptotic proteins and induce both extrinsic and intrinsic apoptotic pathways in cancer cells. Overexpression of prosurvival Bcl-2 family members attenuates HDACi-mediated tumor cell death. HDACis upregulate the BH3-only protein Bim, Bid, and Bmf. HDACis also upregulate DR and TRAIL expression and reduce c-FLIP [104]. Acetylation of non-histone proteins by HDACis may play more important roles in the induction of apoptosis. One such protein is p53, which is acetylated in cancer cells after treatment with HDACi. Acetylated p53 is recruited to the promoters of target genes *Bax* and *Puma* to induce their transcription [33,105]. Acetylated p53 also releases the binding to Bax and allows Bax to translocate to the mitochondria to induce apoptosis [106]. Since p53 mutation frequently occurs in many types of cancers that are responsive to HDACi-induced apoptosis, numerous studies suggest that a p53-independent mechanism may be involved in HDACi-mediated apoptosis [107,108,109]. Recent studies indicate that Ku70 is a new protein that regulates apoptosis via acetylation. Other research groups and we have found that HDACi Trichostatin A, SAHA, Nicotinamide, and Tubacin increase acetylation of Ku70 during the apoptosis induction process [16,62,63,110,111].

## 6. Ku70 Regulates Apoptosis-Related Proteins

Ku70 is a DNA repair factor in the nucleus and forms a heterodimer with Ku80 through the central domain to bind double-strand DNA (dsDNA), which is essential for the classical non-homologous end-joining (c-NHEJ) pathway of double-strand break (DSB) repair [112,113,114].

Many acetylation sites of lysine residues in Ku70, including k539, k542, k544, k553, k556, k317, k331, and k338, have been found. CBP and PCAF have been verified to associate with and acetylate Ku70, which abolishes Ku70 activity for DNA repair and protein interaction [16]. Ku70 is localized not only in the nucleus but is also partially distributed in the cytoplasm [20,115]. Ku70 binds to the cytosolic Bax, Mcl-1, and c-FLIP to increase their stability and protect cells from apoptosis.

### 6.1. Bax

Bax is one of the two effector proapoptotic proteins to control mitochondrial apoptosis. In cancer cells, Bax exists primarily as a monomer in the cytosol. When given enough stimulation, Bax undergoes conformational changes and translocates to the mitochondria where it oligomerizes and induces apoptosis [116]. Ku70 and Bax were found to interact in the cytoplasm and to inhibit Bax activation [20,64,115,117]. Upon Ku70 knockdown or acetylation, Bax dissociates from the Ku70 complex and translocates to the mitochondria [16,117,118,119]. Overexpression of Ku70 blocks apoptotic cell death induced by transfected Bax [120]. Ku70 possesses enzymatic activity to deubiquitinate its partners and to maintain their stability [121]. Bax association with Ku70 promotes Bax deubiquitylation and prevents its degradation by the proteasome [111,122]. The association with Ku70 also keeps Bax away from the mitochondria [121]. However, Ku70 knockdown showed selective apoptotic effects in neuroblastoma cells but not in HEK293 and Hela cells [16,117]. The difference may rely on the amount of Ku70 bound to Bax and the levels of other antiapoptotic proteins in different cancer cells [115].

### 6.2. Mcl-1

Mcl-1 is a key anti-apoptotic protein of the Bcl-2 family and inhibits intrinsic apoptosis by inactivation of the effector protein Bak [123]. Wang et al. [124] discovered that Ku70 interacts with Mcl-1 to deubiquitinate and stabilize it. It has been found that the C-terminal tail (aa 536-609) of Ku70 directly deubiquitinates Mcl-1 protein and removes polyubiquitin chains from ubiquitinated Mcl-1. Ku70 deletion promotes Mcl-1 degradation in vitro and in vivo and elevates the sensitivity of lung cancer cells to the Bcl-2 inhibitor ABT-737 [124].

### 6.3. c-FLIP

Longley et al. found that Ku70 interacts with c-FLIP [125]. Ku70, via its C-terminal region, also binds to and stabilizes c-FLIP protein to prevent its degradation. Acetylation or silencing of Ku70 inhibits its interaction with c-FLIP, leading to c-FLIP degradation by the ubiquitin proteasome system. Downregulation of c-FLIP through acetylation induction of Ku70 could induce apoptosis and/or enhance the sensitivity of cancer cells to chemotherapy [125,126,127].

## 7. Ku70 Acetylation by HDACi Treatment

HDACi enhances Ku70 acetylation, abolishes DNA repair, increases the levels of DNA damage protein H2AX phosphorylation (γ-H2AX) [128,129,130,131,132], and leads to cell cycle arrest and apoptosis [111,133,134]. MS-275 dramatically elevates Ku70 acetylation and Bax activation in medulloblastoma cells [106]. Maspin inhibits HDAC1, increases acetylation of Ku70, reduces Ku70-mediated sequestration of Bax, and results in Bax-mediated apoptosis [63]. Kwok et al. reported that HDAC6 forms a complex with Ku70 and Bax and that depleting HDAC6 or inhibiting HDAC6 by Tubacin increases Ku70 acetylation and Bax-mediated apoptosis [64]. MS-275 and Tubacin have also been reported to induce c-FLIP degradation [125,127]. HDAC6 is a cytoplasmic HDAC [135,136]. MS-275 is a class I HDACi without inhibiting HDAC6. In class I HDACs, HDAC3, not HDAC1 and 2, locates in both the nucleus and the cytoplasm. It seems that inhibition of HDAC3 should account for the cytoplasmic Ku70 acetylation in MS-275-treated cells. All five approved HDACis inhibit HDAC3 and only three inhibit HDAC6. It is possible that the therapeutic effects of the approved five HDACis come from HDAC3 inhibition but needs to be further confirmed. Currently, several HDAC3 inhibitors are being developed, but there is no clinically useful inhibitor available as yet [93,137].

The currently available HDACis act via inhibiting HDAC activity [138]. Recently, we found that a novel glycyrrhetinic acid derivative 10e reduced the protein levels of HDAC3 and HDAC6, increased Ku70 acetylation, released Bax, and degraded c-FLIP [62]. This compound did not inhibit the activity of HDAC in biochemical assays but increased the levels of acetylated Histone 3. In another report, it was shown that proteasome inhibitor Bortezomib reduced the levels of HDAC1-3 expression and induced histone hyperacetylation and apoptosis [139]. Therefore, selectively degrading an HDAC protein could be an alternative approach for cancer therapy.

## 8. Summary

**Figure 2:**
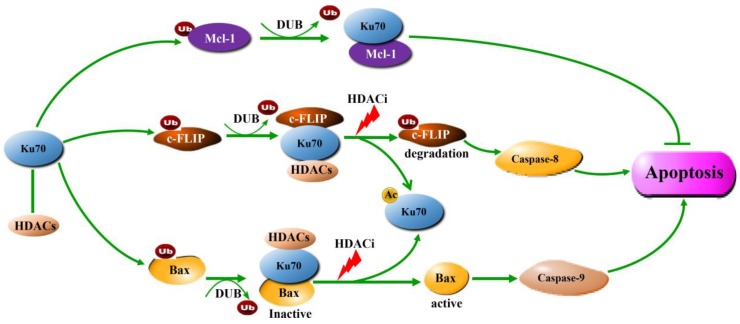
Cytoplasmic Ku70 regulates the activity and stability of apoptosis-related proteins via acetylation. Cytoplasmic Ku70 as a deubiquitinase binds to ubiquitinated Bax, c-FLIP, and Mcl-1 to prevent them from being degraded by proteasomes. After treatment with HDACi, Ku70 is acetylated and dissociates Bax and c-FLIP from the complexes, leading to Bax activation, c-FLIP degradation, and apoptosis initiation. It has been reported that inhibition of HDAC1, HDAC3, HDAC6, SIRT1, SIRT3, or SIRT6 induces Ku70-mediated Bax activation [62,63,64,65,66,67] and that inhibition of HDAC3 and HDAC6 induces Ku70-mediated c-FLIP degradation [62,125].

Although a series of HDAC inhibitors have been on the market and in clinical development, the death mechanism caused by HDACis is not fully understood. Current evidence demonstrates that apoptosis induction is the most effective way for HDACis to kill tumor cells. Acetylation of non-histone protein substrates seems to play more important roles than regulating histone acetylation in the apoptosis induction process. Ku70 is one of the non-histone protein substrates of HDACs. Ku70 regulates DNA repair and protein stability through direct protein–protein interactions. Ku70 functions as a deubiquitinase to bind to ubiquitinated Bax, Mcl-1, and c-FLIP and to stabilize them (Figure 2). HDAC inhibition, especially HDAC3 and HDAC6, causes cytosolic Ku70 acetylation and dissociation of Bax and c-FLIP, resulting in two proapoptotic programs: Bax activation and c-FLIP degradation, which lead to the initiation of both extrinsic and intrinsic apoptotic programs (Figure 2). Overexpression of Ku70 has been found in various human tumors [140]. It has been found that hepatocellular carcinoma (HCC) patients with high Ku70 expression had an obviously poorer prognosis and lower survival rates than those with low Ku70 expression [141]. Since Ku70 blocks apoptosis, which could be reversed by acetylation, it is an attractive therapeutic target for developing HDACi. More studies are needed to determine which HDAC is preferred for binding to Ku70 and for designing selective HDAC inhibitors.

## Figures and Tables

**Table 1:** Non-histone proteins deacetylated by histone deacetylases (HDACs).

Substrate	HDACs	Function of Acetylation	Reference
p53	HDAC1, 2, 3, 6, SIRT1, 2, 7	Increases transcription activity	[33,34,35,36]
NF-κB	HDAC3, SIRT1, 2	Impacts NF-κB translocation and NF-κB-mediated inflammation and transcription	[39,40,59,60]
Hsp90	HDAC3, 6	Promotes chaperone protein degradation	[49,50,51]
STATs	HDAC3	Inhibits JAK2/STAT pathway and NF-kB-mediated inflammatory response	[54,61]
Akt	HDAC3, 6, SIRT1, 2	Decreases Akt phosphorylation and inhibits Akt-mediated pathway	[55,56,57,58]
Ku70	HDAC1, 3, 6, SIRT1, 3, 6	Enhances DNA damage and reduces the stability of Ku70-regulated proteins	[62,63,64,65,66,67]
α-tubulin	HDAC6, SIRT2	Inhibits microtubule assembly and cell motility	[68,69]
c-Myc	HDAC3, 6	Inhibits the transcription ability and decreases c-Myc expression	[70,71]
E2F1	HDAC1	Increases DNA-binding affinity	[72]
HIF-1α	HDAC2, 4, 5, 7	Regulates its degradation	[73,74,75,76]
ER	HDAC1	Increases cell proliferation	[77]
β-catenin	HDAC6	Inhibits its nuclear translocation	[78]
GATAs	HDAC3, 4, 5	Affects erythroid differentiation	[79,80]
FoxOs	SIRT1	Decreases DNA-binding affinity and reduces transcription activity	[81]

**Table 2:** The HDAC inhibitors approved for clinic use.

HDAC Inhibitor	Chemical Class	Structure of the Compound	HDAC Class Inhibited
Vorinostat (SAHA)	Hydroxamic acid	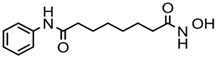	Pan-HDACs
Romidepsin (FK228)	Cyclic peptide	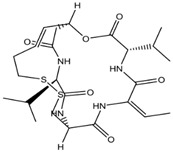	Class I
Belinostat (PXD-101)	Hydroxamic acid	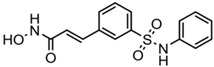	Pan-HDACs
Panobinostat (LBH-589)	Hydroxamic acid	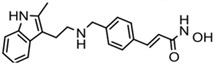	Pan-HDACs
Chidamide	Benzamide	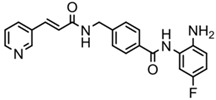	Class I, HDAC10
